# Combination of Collagen-Chitosan Hydrogel and Injectable Platelet-Rich Fibrin as a Biomaterial for Bone Regeneration: Characterization and Growth Factor Release Pattern

**DOI:** 10.1055/s-0045-1809144

**Published:** 2025-05-22

**Authors:** Dharmmesti Anindita Wijayanti, Gusti Ngurah Komang Agus Wirajaya, Nuansa Hanum Pratiwi, Vincensia Maria Karina, Kwartarini Murdiastuti

**Affiliations:** 1 Periodontics Specialist Study Program, Faculty of Dentistry, Universitas Gadjah Mada, Yogyakarta, Indonesia; 2 Department of Periodontics, Faculty of Dentistry, Universitas Gadjah Mada, Yogyakarta, Indonesia

**Keywords:** scaffold, collagen-chitosan hydrogel, injectable platelet-rich fibrin, FTIR, PDGF-AB, TGF-β1

## Abstract

**Objective:**

The release of growth factors in injectable platelet-rich fibrin (I-PRF) exhibits a peak within 24 hours and subsequent decline by day 10, underscoring immediate application, limiting its effectiveness in alveolar bone repair. In order to enhance its regenerative potential, I-PRF can be combined with biomaterial scaffolds such as collagen-chitosan hydrogels, which mimic the extracellular matrix and support tissue regeneration. This combination has been shown to enhance cellular signaling and tissue repair. This study aimed to analyze the characterization of collagen-chitosan hydrogels with I-PRF and determine the growth factor release pattern that occurs after mixing.

**Materials and Methods:**

Collagen-chitosan hydrogels were prepared and combined with I-PRF at a 1:1 ratio. The structural characterization of these hydrogels, both with and without I-PRF, was performed using Fourier transform infrared spectroscopy (FTIR), enabling the comparison of absorption bands. Furthermore, the release profiles of transforming growth factor-beta 1 (TGF-β1) and platelet-derived growth factor AB (PDGF-AB) were assessed in two experimental groups: The first group consisted of I-PRF alone, while the second group comprised of I-PRF combined with collagen-chitosan hydrogels. Growth factor release was evaluated at multiple time points (days 1, 3, 5, 7, 9, 11, 13, 15, and 17) using enzyme-linked immunosorbent assay. The resulting absorbance values were converted into concentration measurements (pg/mL) using a standard calibration curve. Statistical analysis was conducted using two-way analysis of variance followed by a *post hoc* least significant difference test.

**Results:**

FTIR analysis demonstrated that the functional groups present in the collagen-chitosan hydrogel remained unchanged following the incorporation of I-PRF, confirming the formation of physical rather than chemical bonds. Subsequent analysis revealed statistically significant differences in the release patterns of TGF-β1 and PDGF-AB between the two groups ( *p* < 0.05). The combination of collagen-chitosan hydrogel and I-PRF exhibited a more stable and sustained release profile from day 1 to day 17.

**Conclusion:**

The combination of I-PRF with collagen-chitosan hydrogels does not alter the fundamental chemical structure of the scaffold. However, this combination does influence the controlled release of growth factors. This finding indicates that the synergistic interaction between collagen and chitosan enhances the hydrogel's properties, suggesting its potential as a promising biomaterial for use as a scaffold in bone regeneration.

## Introduction

The Global Burden of Diseases Study reports a worldwide prevalence of periodontitis of 1.1 trillion cases. 1 Delayed periodontitis treatment has the potential to result in tooth loss, which can lead to further general health complications. 2 Periodontal disease is the leading cause of tooth loss worldwide.

A variety of regenerative surgical procedures have been studied to support the regeneration of certain periodontal tissues, one of which is the guided tissue regeneration/guided bone regeneration (GTR/GBR) strategy. 3 Tissue regeneration technology is being developed extensively with tissue engineering, that is, regeneration that involves a combination of several elements, including cell regeneration, biomaterials such as scaffolds or membranes, and biochemistry such as cell signaling molecules or growth factors. 4 GTR/GBR requires membrane or scaffold components that act as an extracellular matrix (ECM) component, so it is necessary to add growth factors that play a role in the healing and regeneration of periodontal tissues. 5

Growth factors are a critical component of tissue regeneration, and they are present in high concentrations within platelet concentrates. Platelet concentrates, defined as autologous biological blood-derived products, are composed of fibrin-forming proteins and bioactive components derived from plasma and platelets. These components possess the capacity to naturally form a three-dimensional structure. 6 Injectable platelet-rich fibrin (I-PRF) is one of the formulations of PRF with a liquid consistency that is prepared with a modified time and lower centrifugation speed of 700 revolutions per minute (rpm) for 3 minutes, resulting in a higher concentration of growth factor and greater regenerative potential. 7 A comparison of the effects of I-PRF and platelet-rich plasma (PRP) in previous studies revealed a significant increase in the release of growth factors, including transforming growth factor-beta (TGF-β), platelet-derived growth factor (PDGF), messenger ribonucleic acid, fibronectin, and type 1 collagen. The release pattern of these growth factors in I-PRF has been observed previously and exhibits an initial increase within the first 15 minutes, followed by a subsequent peak within the first 8 hours of the day. This pattern subsequently demonstrates a decline, reaching a nadir on the 10th day. However, it is imperative to employ I-PRF promptly due to its susceptibility to rapid degradation when in liquid consistency. 7 8 Consequently, the optimal application of I-PRF is hindered in the treatment of alveolar bone damage. Nevertheless, its combination with diverse biomaterials, such as scaffolds, has been shown to enhance tissue healing capacity. 9

The combination of collagen and I-PRF has been demonstrated to be a safe and effective approach for regulating the rate of release of growth factors released by I-PRF. 10 However, collagen is characterized by its rapid degradation, which can lead to alterations in its shape and size. To address this challenge, the incorporation of a stabilizing agent, such as chitosan, is necessary to enhance the collagen's resistance to degradation. The combination of collagen and chitosan is expected to enhance mechanical strength. 11 The presence of chitosan, a biomacromolecule, in hydrogel-shaped scaffolds has been shown to possess antioxidant and antibacterial properties, in addition to its ability to activate the immune response. 12 Hydrogel has demonstrated its potential as a microenvironment provider for tissue regeneration and drug delivery systems. The hydrogel's resemblance to an ECM is a key factor in its consideration as a scaffold material, as it facilitates cell proliferation, vascularization, and survival. 13 The I-PRF with collagen-chitosan hydrogel is a novel material that has the potential to be utilized as a periodontal regenerative treatment. Therefore, a bond test is necessary to ascertain the functional groups formed as a result of the interaction between I-PRF, the active ingredient that triggers periodontal tissue regeneration, and a collagen-chitosan hydrogel complex that serves as a scaffold. The bond test aims to establish physical bonding between these materials. The Fourier transform infrared (FTIR) method is to be used to detect the functional groups formed between I-PRF and collagen-chitosan hydrogel. The FTIR test is a method that can detect intermolecular interactions that occur between two materials. 14 The FTIR test will produce a graph showing the absorption band to determine the chemical functional groups present in the material. The intensity of specific absorption bands within the spectrum is indicative of the functional groups present in the material under investigation. 15 The combination of collagen-chitosan hydrogel with a collagen-chitosan ratio (25/75) has been found to meet the established criteria (pH, viscosity, and swelling) and functions as a reservoir growth factor by binding and retarding the release of active ingredients. The utilization of hydrogel is anticipated to prolong the release of growth factors from I-PRF. 16 17

## Materials and Methods

### Ethical Clearance

The ethics have been ratified by the Ethics Commission of the Faculty of Dentistry and RSGM Prof. Soedomo, Gadjah Mada University (154/UN1/KEP/FKG-RSGM/EC/2023 and 223/UN1/KEP/FKG-RSGM/EC/2023). All methods are carried out in accordance with relevant guidelines and regulations.

### Preparation of I-PRF

Blood was drawn from male donors aged 20 to 30 years with normal platelet counts and no systemic diseases or long-term medication use. Female donors were excluded due to the potential influence of hormonal factors. A total volume of 10 mL of blood was collected in a plastic centrifugation tube (Iwaki). Centrifugation was performed at 700 rpm for 3 minutes, thereby separating the blood into three layers: a yellow layer, a buffy coat layer, and a red layer. The red layer, which contains I-PRF, is obtained through aspiration using a 3-mL syringe and an 18G needle on the buffy coat layer.

### Collagen-Chitosan Hydrogel Mixture

The hydrogel is composed of a blend of two pharmaceutical-grade ingredients: collagen from fish (chitosan) and collagen. The formulation of the collagen-chitosan hydrogel (25/75) consists of 0.5 g of collagen, 1.5 g of chitosan, and 2 g of hydroxypropyl methylcellulose (HPMC). Chitosan is introduced into a glass beaker containing 100 mL of water and stirred at a speed of 3,000 rpm using a magnetic stirrer until it is fully dispersed. Subsequently, 1% acetic acid is incorporated. The preparation of the HPMC powder and collagen entails their precise weighing on a digital scale. The collagen powder is then introduced into the chitosan solution, where it is stirred at 3,000 rpm until the mixture is homogeneous. Subsequently, the HPMC powder is added to the solution using a homogenizer at the same stirring rate. Following homogenization, the hydrogel is left for 15 minutes at room temperature and subsequently placed in a refrigeration machine. 16

### Mixing Collagen-Chitosan Hydrogel with I-PRF

The collagen-chitosan hydrogel was mixed with I-PRF at a volumetric ratio of 1:1, with the volume of I-PRF being 1 mL. This mixture was prepared using a micropipette.

### Infrared Spectral Analysis of Collagen-Chitosan Hydrogel, I-PRF, and Collagen-Chitosan Hydrogel with I-PRF

Collagen-chitosan hydrogel, I-PRF, and collagen-chitosan hydrogel with I-PRF were deposited in an FTIR sample container in amounts of up to one drop. Subsequently, analysis of each material using FTIR was carried out on a sample with a wave range of 4000 to 400 cm ^**−** 1^ with a resolution of 2 cm ^**−** 1^ to 16 scans. 18

### Analysis of TGF-β1 and PDGF-AB Emission Rate

Culture media, a combination collagen-chitosan hydrogel and I-PRF, was immersed in a culture media solution at a ratio of 1:3 and subsequently incubated at 37°C. At predetermined intervals of 1, 3, 5, 7, 9, 11, 13, 15, and 17 days, the soaking solution was retrieved and utilized for further analysis. Each sampling was carried out in a sterile barrier system and placed in a microtube that had been marked. The microtubes were stored in a freezer set at −80°C. The well of each microtube was then refilled with culture media, and the microtubes were incubated at 37°C. This cycle is repeated for the subsequent day of collection until the 17th day. 19

### Statistical Analysis

**Fig. 1:**
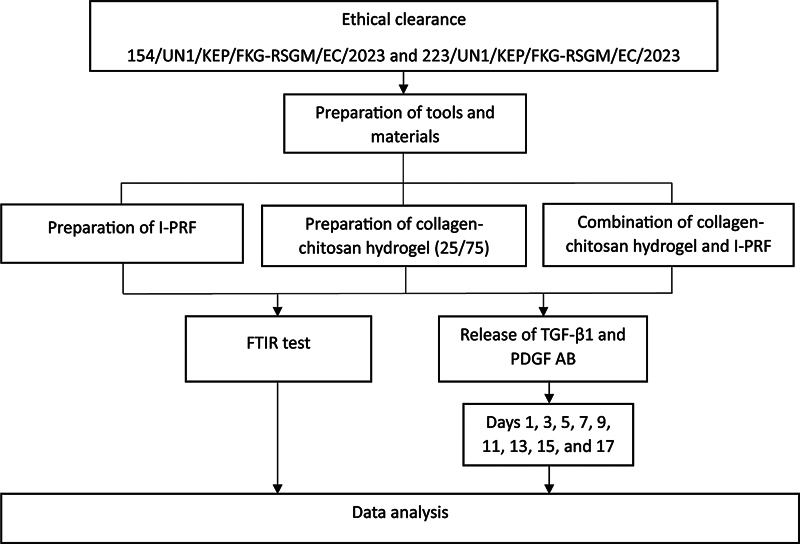
Research road map.

The normality test used was the Shapiro–Wilk test, followed by the Levene test for homogeneity. Subsequently, a two-way analysis of variance test was conducted with a 95% confidence level to ascertain the effect of adding I-PRF to collagen-chitosan hydrogel on the release pattern of TGF-β1 and PDGF-AB. To further refine the findings, a *post hoc* least significant difference test was conducted to assess the statistical significance of any observed differences between the designated observation time groups. The research road map presented in Fig. 1 .

## Results

### Fourier Transform Infrared Spectroscopy

The results demonstrated a comparison of the infrared spectral picture of I-PRF, collagen-chitosan hydrogel, and the mixing results of I-PRF with collagen-chitosan hydrogel. The resulting image indicated that all materials exhibited a similar absorption band peak, suggesting that each material retained its existing functional group.

The absorption band on the collagen-chitosan hydrogel manifests the combined functional groups of collagen and chitosan. The absorption band at 3317.74 cm ^**−** 1^ , which corresponds to the peak of the band, indicates the presence of hydroxyl functional groups (–OH) and N-H stretching. The absorption band at 2114.18 cm ^**−** 1^ corresponds to the stretching C = C functional group. The absorption band at 1635.61 cm ^**−** 1^ corresponds to the N-H bending functional group. The absorption band at 1415.03 cm ^**−** 1^ also corresponds to the N-H bending functional group.

The absorption band on the I-PRF presents the functional group of the I-PRF. The absorption band at 3285.33 cm ^**−** 1^ is indicative of the presence of hydroxyl functional groups (–OH) and N-H stretching. The absorption band at 2111.51 cm ^**−** 1^ is indicative of a stretching C = C = C functional group. The absorption band at 1636.32 cm ^**−** 1^ corresponds to the N-H bending functional group. The absorption band at 1549.53 cm ^**−** 1^ also corresponds to the N-H bending functional group. The absorption band at 1457.03 cm ^**−** 1^ also corresponds to the N-H bending functional group. Finally, the peak at 1397.55 cm ^**−** 1^ serves as an additional indicator of the N-H bending functional group.

**Fig. 2:**
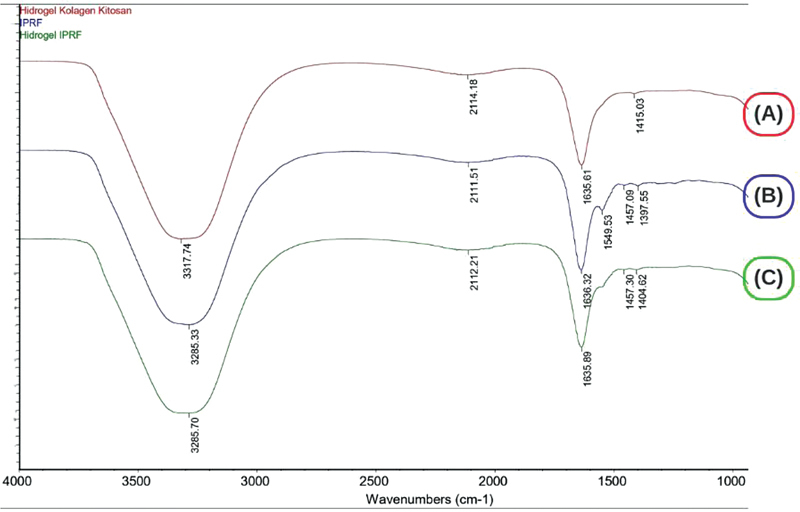
Spectra Fourier transform infrared spectroscopy (FTIR) collagen-chitosan hydrogel ( **A** ), injectable platelet-rich fibrin (I-PRF) ( **B** ), and collagen-chitosan hydrogel + I-PRF ( **C** ).

The absorption band of the collagen-chitosan hydrogel mixed with I-PRF demonstrates the combined functional groups of each constituent ingredient. The absorption band at 3285.70 cm ^**−** 1^ in the collagen-chitosan hydrogel + I-PRF indicates the presence of hydroxyl functional groups (–OH) and N-H stretching, accompanied by a shift in the wave peak. These functional groups are also present in pure collagen and chitosan hydrogels. The shift in the wave peak from 3317.74 to 3285.70 cm ^**−** 1^ was observed in the collagen-chitosan hydrogel following the addition of I-PRF. This shift is attributed to the interaction between collagen-chitosan hydrogels, which is a hallmark of the presence of hydroxyl and N-H groups ( Fig. 2 ).

### Growth Factors Release Pattern

A study was conducted to investigate the impact of a combination of collagen-chitosan hydrogel and I-PRF on the release pattern of TGF-β1 and PDGF. Test data was collected on two distinct samples to observe the growth factors release pattern by groups A, which includes I-PRF, and B, which includes combination collagen-chitosan hydrogel and I-PRF. The experimental design encompassed a 17-day observation period, subdivided into nine distinct time points: days 1, 3, 5, 7, 9, 11, 13, 15, and 17.

**Fig. 3:**
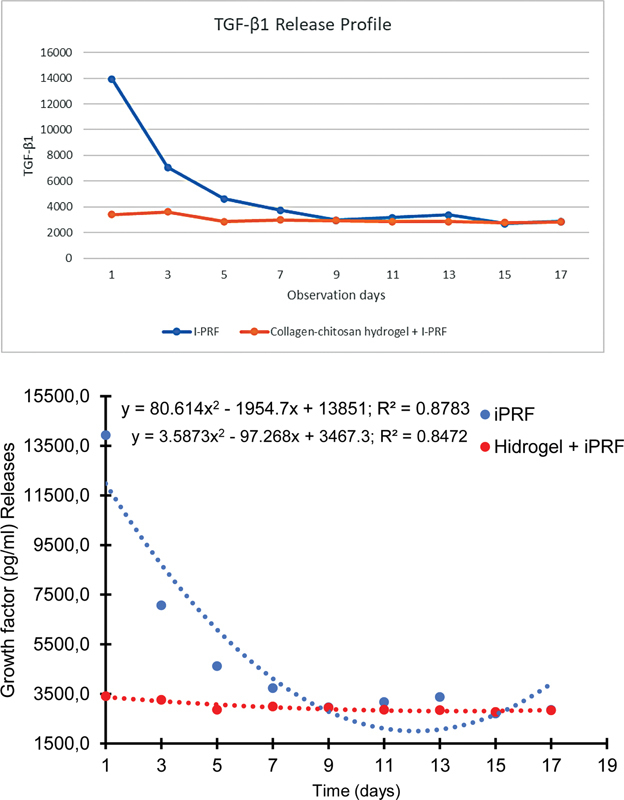
Diagram of transforming growth factor-beta 1 (TGF-β1) release (pg/mL).

The mean findings from the observation of TGF-β1 levels based on group A and group B are displayed in Table 1 and Fig. 3 . According to the observed data, there was a discrepancy in the levels of release from the first day of observation between group A and group B. Group A demonstrated a higher release compared to group B on the first day. On day 3, group A exhibited a reduction of over 50% from the initial level, whereas group B demonstrated a slight decline. During the subsequent observation period, group A demonstrated a decline in TGF-β1 concentration, reaching a decreasing level by day 15, with a slight increase observed on day 17. The analysis of polynomial tests in group A further revealed a significant difference in timings within the group (bold *p* -Values).

**Table 1:** Average and standard deviation of TGF-β1 release rate based on observation and treatment time

Observation time	*n*	Group A	Group B	*p* -Value
Day 1	3	13,934.26 ± 459.09	3,412.94 ± 37.88	**0.000**
Day 3	3	7,074.20 ± 133.32	3,625.21 ± 54.43	**0.000**
Day 5	3	4,620.47 ± 74.45	2,861.64 ± 11.88	**0.000**
Day 7	3	3,731.78 ± 80.54	2,994.68 ± 32.18	**0.000**
Day 9	3	2,966.51 ± 31.82	2,945.66 ± 56.95	**0.832**
Day 11	3	3,173.36 ± 49.76	2,861.74 ± 19.57	**0.003**
Day 13	3	3,389.68 ± 39.99	2,846.38 ± 19.18	**0.000**
Day 15	3	2,713.13 ± 16.54	2,773.96 ± 7.90	**0.537**
Day 17	3	2,857.09 ± 39.18	2,841.38 ± 21.98	**0.197**

Abbreviation: TGF-β1, transforming growth factor-beta 1. Bold *p* -Values < 0.05.

**Fig. 4:**
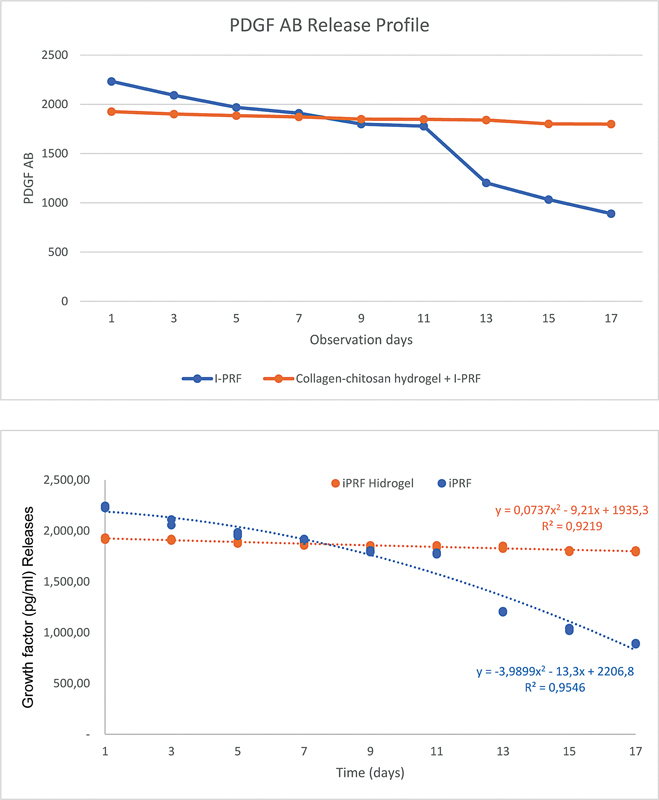
Diagram of the standard deviation line of the discharge concentration of platelet-derived growth factor AB (PDGF-AB).

In contrast, group B exhibited a consistently constant pattern of TGF-β1 release from day 1 to day 17. This is in contrast to group A, which exhibited a propensity to reduce the release pattern as the overall discharge concentration levels increased throughout the observation day. Group B displayed lower levels of total release concentration. Group B exhibited a consistent pattern and demonstrated the capacity to regulate the release of TGF-β1.

The release pattern data for PDGF-AB are presented in Table 2 and further corroborated by Fig. 4 . In a manner analogous to TGF-β1, group B exhibited the capacity to regulate a stable release pattern from the first day. The collagen-chitosan hydrogel demonstrated the capacity to sustain the release of growth factors until day 17, in contrast to the I-PRF group, which exhibited the highest release of growth factors on day 1, followed by a decline until day 11, with a significant decrease from days 13 to 17.

**Table 2:** The average value and standard deviation of the concentration of PDGF-AB release were based on the observation time of the two groups

Observation time	*n*	Group A	Group B	*p* -Value
Day 1	3	2,234.13 ± 11.65	1,927.07 ± 11.37	**0.000**
Day 3	3	2,093.59 ± 31.59	1,901.07 ± 7.02	**0.000**
Day 5	3	1,969.10 ± 18.35	1,886.45 ± 11.77	**0.000**
Day 7	3	1,911.87 ± 8.02	1,873.16 ± 15.83	**0.072**
Day 9	3	1,800.97 ± 8.48	1,850.50 ± 11.16	**0.005**
Day 11	3	1,779.81 ± 8.74	1,848.81 ± 12.03	**0.000**
Day 13	3	1,203.46 ± 5.76	1,842.14 ± 14.46	**0.000**
Day 15	3	1,033.16 ± 13.69	1,802.34 ± 8.33	**0.000**
Day 17	3	891.74 ± 4.69	1,800.27 ± 7.48	**0.000**

Abbreviation: PDGF-AB, platelet-derived growth factor AB. Bold *p* -Values < 0.05.

## Discussion

The scaffold plays a pivotal role in the tissue regeneration process. The ideal scaffold must possess the following properties: biodegradable, biocompatible, bioactive, osteoinductive, and osteoconductive characteristics. 20 The clinical requirements for scaffolds include a stable structure, the ability to support cell migration, the capacity to act as a reservoir for growth factors to stimulate vascularization and cell regeneration, and scaffold resorption or degradation in parallel with the regeneration process. 21

### Fourier Transform Infrared Spectroscopy

The absorption band of the collagen-chitosan hydrogel mixed with I-PRF demonstrates the combined functional groups of each constituent ingredient. The absorption band at 3285.70 cm ^**−** 1^ in the collagen-chitosan hydrogel + I-PRF indicates the presence of hydroxyl functional groups (–OH) and N-H stretching, accompanied by a shift in the wave peak. These functional groups are also present in pure collagen and chitosan hydrogels. The shift in the wave peak from 3317.74 to 3285.70 cm ^**−** 1^ was observed in the collagen-chitosan hydrogel following the addition of I-PRF. The presence of hydroxyl and N-H groups is attributed to the interaction between collagen-chitosan hydrogels. As previously reported by Devernois and Coradin, 22 peaks between 3500 and 3000 cm ^**−** 1^ are characteristic of both collagen and chitosan, corresponding to the N-H and O-H vibrations of proteins and polysaccharides.

The maximum of the absorption band at wave number 2112.21 cm ^**−** 1^ of collagen-chitosan hydrogel + I-PRF signifies the existence of a stretching C = C functional group. This functional group has also been identified in the infrared spectra of pure chitosan and collagen hydrogels. According to Devernois and Coradin, 22 the absorption peak between 2200 and 2000 cm ^**−** 1^ is a characteristic of chitosan and does not appear in collagen. This finding aligns with the results of comparative studies, which reported a peak of absorption at 2115.53 cm ^**−** 1^ as the background of the C = C stretching cluster owned by I-PRF. 23

An N-H bending functional group was identified at the wave number 1635.89 cm ^**−** 1^ in the collagen-chitosan hydrogel + I-PRF. This functional group was also seen in I-PRF in the wave numbers 1636.32 and 1635.61 cm ^**−** 1^ in the collagen-chitosan hydrogel. An additional finding of the current study was the detection of an N-H functional group in PRF in conjunction with chitosan, which was previously identified in other research. 24 Moreover, the aforementioned research indicated that the amide I functional group in collagen and chitosan is present within the wave range of 1750 to 1550 cm ^**−** 1^ . The amide I functional group in collagen and chitosan is present within the wave range of 1750 to 1550 cm ^**−** 1^ . 25

The wave number is 1551.72 cm ^−1^ in collagen-chitosan hydrogel + I-PRF, indicating the presence of an N-H bending functional group, which is also observed in I-PRF at the wave number 1549.53 cm ^−1^ . This finding aligns with the observations reported in the research conducted by Mirhaj et al. 26 This research utilized comparative analysis, and the results indicated a peak absorption of 1546 cm ^−1^ in PRF, attributed to the presence of the amide II functional group.

The analysis of the collagen-chitosan hydrogel in combination with I-PRF indicates the presence of an N-H bending functional group at specific wave numbers: 1457.30 and 1404.62 cm ^−1^ . This finding is consistent with the literature on I-PRF, which reports the presence of this functional group at wave numbers 1457.09 and 1397.55 cm ^−1^ . Moreover, the collagen-chitosan hydrogel displays the same N-H bending functional group at a specific wave number of 1415.03 cm ^−1^ . This finding aligns with the research conducted by Sánchez-Cid et al, 25 which also indicates that the amide II functional group in collagen and chitosan is located within the wave range of 1550 to 1250 cm ^−1^ . Another study also stated that the wave number 1442 cm ^−1^ is the amide II functional group in PRF.

A thorough analysis of the experimental results and a comparative analysis of the various materials revealed that there was an absence of any novel functional groups identified following the amalgamation of collagen-chitosan hydrogel with I-PRF. Although a slight shift in the peak of the wave number was observed, it remained within the range of the number of functional groups. This shift is attributed to the dynamic nature of the atom. 27 It is noteworthy that all the peaks of the absorption band correspond to the functional groups inherent in each of these materials. This finding aligns with the conclusions of several studies, including that of Diaz-Gomez et al, 28 which reported that the immersion of nanofibers in blood material (PRP) exhibited no change in the absorption band peak before and after soaking. Conversely, Kai et al 29 asserted that the absence of a new absorption band peak in the FTIR test indicates the absence of new substance formation in the material postmixing. The absence of a new substance indicates that no chemical bond has been formed between the substances involved, suggesting that the interaction is limited to physical interactions.

## Growth Factors Release

A notable disadvantage of I-PRF is the inconsistent release of growth factors, which impedes its effectiveness in periodontal regenerative treatment. Additionally, the liquid consistency of the substance imposes limitations on its shelf life, consequently diminishing its efficacy in the wound healing process. In this study, the observation of growth factor release was conducted over a period of 17 days, which is consistent with the proliferation and differentiation stage in periodontal tissue regeneration, which can extend to 15 days. The release of TGF-β1 in group A exhibited a high release pattern on day 1, followed by a significant decrease on the subsequent day. In contrast, group B exhibited a more moderate and consistent release pattern, remaining relatively stable from day 1 until day 17. These findings suggest that group B has a greater potential to regulate the release of growth factors more effectively than group A. The amount of protein released remained below 60% until day 10, demonstrating the exceptional capacity of the PRF-gelatin chitosan hydrogel combination to regulate protein release. 12 TGF-β is linked to the process of healing and has the ability to enhance bone regeneration. During the process of osteoblast differentiation, the TGF-β1 receptor binds to active TGF-β and interacts with the TGF-β2 receptor to enhance osteoblastogenesis, osteoblast differentiation, expression of alkaline phosphatase, and type I collagen. 30 The proliferation and differentiation stages involved in the periodontal tissue regeneration process can extend beyond 15 days. 31

A significant decrease in PDGF-AB release was observed on day 13, with a notable decline in the combination collagen-chitosan hydrogel and I-PRF. The mean value remained stable, exhibiting a minimal decline. The depletion of fibrinogen in I-PRF results in the absence of any inhibitory effect on the release rate of growth factors that occurred starting from day 11. The gradual and controlled release of both growth factors was shown in the combination of collagen-chitosan hydrogel and I-PRF, effectively slowing the release rate of the growth factors. This controlled release continued throughout the entire observation period. The formation of the hydrogel is a result of the interaction between collagen and chitosan, which are the two elements with the appropriate composition. Chitosan has the ability to modify bond structures through mechanical means. These bonds exhibit strong hydrophilicity and polarity. Furthermore, chitosan can undergo synthesis to create interconnected pores with an optimal size and specific characteristics that facilitate cell infiltration, new tissue growth, nutrient transport, and the diffusion of active compounds. The selection of hydrogel-forming components is compatible with cells and tissues, promoting cell adhesion and sustained proliferation within the hydrogel's pores. This release pattern is expected to continue aiding in the wound healing process, specifically during the inflammatory phase (days 0–5), the proliferation phase (days 3–14), and the remodeling or maturation phase that lasts for several weeks. 32 The findings of this study demonstrate that the combination chitosan and collagen in a hydrogel can serve as an effective drug delivery system, capable of extending the duration of drug or therapeutic chemical effects. 33

In a statistical analysis, the significance of a set of data is influenced by several factors, such as a small sample size, insufficient variation between groups, or high variability. These factors have the potential to affect specific groups that demonstrate substantial outcomes. However, the present study is concerned with the release pattern of growth factors observed with the addition of collagen-chitosan hydrogel as a scaffold, with the objective of addressing the research question. The observed pattern differences suggest that the combination of collagen-chitosan hydrogel and I-PRF results in a linear pattern, whereas I-PRF without collagen-chitosan hydrogel exhibits a parabolic pattern. This similarity in values at certain time points may contribute to the statistical analysis yielding nonsignificant results. Nevertheless, based on the observed pattern, the consistent release of growth factors in combination of collagen-chitosan hydrogel and I-PRF demonstrates significant potential, suggesting an improvement in periodontal wound healing efficacy. It is important to note that the present study is still at the *in vitro* stage, and therefore requires further research in the *in vivo* phase.

## Conclusion

The combination of collagen-chitosan hydrogel with I-PRF preserves the absorption band of the functional group, indicating that no new substance is formed. This suggests that the interaction between the two materials is predominantly physical in nature, rather than involving chemical bonds. The presence of I-PRF in collagen-chitosan hydrogel has been shown to affect the stability of the release pattern of TGF-β1 and PDGF-AB. The synergy between collagen and chitosan enhances the properties of the hydrogels, demonstrating their potential as effective biomaterials for use in periodontal regeneration.
